# Targeted silencing of *GNAS* in a human model of osteoprogenitor cells results in the deregulation of the osteogenic differentiation program

**DOI:** 10.3389/fendo.2024.1296886

**Published:** 2024-05-17

**Authors:** Francesca Marta Elli, Deborah Mattinzoli, Masami Ikehata, Francesca Bagnaresi, Maria A. Maffini, Giulia Del Sindaco, Angela Pagnano, Camilla Lucca, Piergiorgio Messa, Maura Arosio, Giuseppe Castellano, Carlo M. Alfieri, Giovanna Mantovani

**Affiliations:** ^1^ Endocrinology Unit, Fondazione IRCCS Ca’ Granda Ospedale Maggiore Policlinico, Milan, Italy; ^2^ Department of Nephrology, Dialysis and Renal Transplantation, Fondazione IRCCS Ca’ Granda Ospedale Maggiore Policlinico, Milan, Italy; ^3^ Department of Clinical Sciences and Community Health, University of Milan, Milan, Italy

**Keywords:** *GNAS*, ectopic bone, mesenchymal stem cells, osteogenesis, human cell model

## Abstract

**Introduction:**

The dysregulation of cell fate toward osteoprecursor cells associated with most *GNAS*-based disorders may lead to episodic *de novo* extraskeletal or ectopic bone formation in subcutaneous tissues. The bony lesion distribution suggests the involvement of abnormal differentiation of mesenchymal stem cells (MSCs) and/or more committed precursor cells. Data from transgenic mice support the concept that *GNAS* is a crucial factor in regulating lineage switching between osteoblasts (OBs) and adipocyte fates. The mosaic nature of heterotopic bone lesions suggests that *GNAS* genetic defects provide a sensitized background for ectopic osteodifferentiation, but the underlying molecular mechanism remains largely unknown.

**Methods:**

The effect of *GNAS* silencing in the presence and/or absence of osteoblastic stimuli was evaluated in the human L88/5 MSC line during osteodifferentiation. A comparison of the data obtained with data coming from a bony lesion from a *GNAS*-mutated patient was also provided.

**Results:**

Our study adds some dowels to the current fragmented notions about the role of *GNAS* during osteoblastic differentiation, such as the premature transition of immature OBs into osteocytes and the characterization of the differences in the deposed bone matrix.

**Conclusion:**

We demonstrated that our cell model partially replicates the *in vivo* behavior results, resulting in an applicable human model to elucidate the pathophysiology of ectopic bone formation in *GNAS*-based disorders.

## 1 Introduction

The parathyroid/parathyroid-related protein hormone–α subunit of the stimulatory G protein–cyclic AMP (PTH/PTHrP-Gsα-cAMP) signaling pathway is a well-known regulator of calcium metabolism and postnatal bone homeostasis. It regulates osteogenesis through a direct and indirect interrelationship with multiple signaling pathways, such as transforming growth factor-beta/bone morphogenic protein, Wnt-β-catenin, and fibroblast growth factors (FGFs) (1–3). Part of this regulation is mediated through Runt-related transcription factor 2 (*RUNX2*), which is the master gene that switches the strictly regulated multistep molecular pathway of osteodifferentiation by supporting the transduction of osteogenic signals from the extracellular environment to the nucleus (4–6).

The dysregulation of the cell fate determination, associated with a triggering event in precursor cells with the potential to differentiate into bone, leads to the development of heterotopic ossification (HO). The presence of HO characterizes different rare and genetically determined human disorders. In particular, most *GNAS*-based disorders share the common feature of episodic *de novo* formation of islands of extraskeletal, ectopic, qualitatively normal bone in the skin and subcutaneous fat (7–10).

The complex imprinted *GNAS* locus encodes Gsα, its primary transcript, the extra-large variant of Gsα (XLαs), the neuroendocrine protein 55 (*NESP55*), and additional sense and antisense non-translated transcripts. Genetic and/or epigenetic defects determining a partial deficiency of Gsα lead to resistance to the action of the PTH hormone, which is the hallmark of pseudohypoparathyroidism (PHP), and the alteration of the PTH/PTHrP-Gsα-cAMP signaling pathway (11–16).

Different subtypes of PHP have been described based on the existence of additional clinical features, such as resistance to other hormones (TSH, GHRH, and gonadotropins), obesity, and Albright’s hereditary osteodystrophy (AHO), which includes short stature, rounded face, brachydactyly, ectopic ossifications, and cognitive impairment (13, 14, 17–21). In AHO, HO usually presents during late childhood or adulthood and is restricted to the cutaneous and subcutaneous tissue. In contrast, progressive osseous heteroplasia (POH) presents during infancy, with dermal and subcutaneous ossifications that progress during childhood into the skeletal muscle and deep connective tissues (8–10, 22).

PHP-related HOs occur predominantly through an intramembranous process; unlike endochondral ossification, cartilage is absent (7). The tissue distribution of HO lesions suggests that the pathogenesis involves abnormal differentiation of mesenchymal stem cells (MSCs, also known as multipotent mesenchymal stromal cells) and/or more committed precursor cells that are present in skin, subcutaneous fat, muscle, tendon, and ligament tissue; however, the underlying molecular mechanism remains largely unknown (7, 23). The mosaic nature of HO in *GNAS*-related disorders suggests that *GNAS* defects provide a sensitized background for ectopic osteoblast (OB) differentiation (24, 25).

Data from transgenic mice support the concept that *GNAS* is crucial in regulating the lineage switching between OB and adipocyte fates. Its role may prevent the bone formation in tissues where bone should not form. Transcriptional changes in *Gnas* +/− mouse soft tissue stem cells are accompanied by accelerated OB differentiation, enhanced expression of osteogenic markers, and development of subcutaneous HO *in vivo* (26–28). At the molecular level, *GNAS* inhibits osteodifferentiation through the proteolytic degradation of *RUNX2*, a transcription factor fundamental in differentiating bone precursor cells into OBs. *GNAS* downregulation leads to increased expression of *RUNX2*, resulting in the production of bone-differentiating proteins (29, 30). The process of HO in rare genetic disorders such as AHO/PHP/POH and the pathophysiological mechanisms by which alterations of PTH/PTHrP-Gsα-cAMP signaling led to ectopic bone formation are not fully understood; to date, investigations have been mainly conducted in mouse models (26, 31, 32). Data describing the molecular and functional characterization of human HO-forming cell lineages are missing, and no robust human cell model has been generated. The few attempts to study the role of *GNAS* in HO formation in humans used antisense oligonucleotides in bone marrow-derived primary MSCs from the iliac crest of healthy donors and the SQ22,536 cyclic adenosine monophosphate (cAMP) inhibitor in adipose-derived primary MSCs from liposuction procedures (25, 33).

This paper presents a deep characterization of a human MSC line osteodifferentiation, highlighting the resulting specific alterations when subjected to *GNAS* silencing in the presence or absence of external OB-inducing stimuli. Our work highlights previously undescribed aspects of ectopic OB differentiation in AHO/PHP, such as the premature transition of immature OBs to the osteocyte (OS) phenotype and the characterization of qualitative and quantitative differences in the bone extracellular matrix (ECM) produced by *GNAS*-silenced OB. Finally, we investigated the behavior of cells from a surgically removed lesion of an extraskeletal bone from a young PHP-mutated girl; our MSCs partially replicated the behavior observed in patient-derived cells, demonstrating that our *GNAS*-silenced MSCs were a reliable cell model of human OB precursors, useful to elucidate the molecular pathophysiology of ectopic bone formation in *GNAS*-based disorders.

## 2 Materials and methods

### 2.1 L88/5 cell cultures, GNAS silencing, and osteogenic differentiation

The L88/5 human MSC line (RRID: CVCL 6838, permanent Simian virus 40-transformed human stromal cell lines established and kindly provided by Dr. Thalmeier) was cultured in basal medium containing RPMI-1640 with 10% fetal bovine serum (FBS) and 1% penicillin/streptomycin (both from Sigma-Aldrich, RRID: SCR_008988) and medium was replaced every 2–3 days. The OB differentiation was promoted by the administration of the OB-Inducer Reagent containing dexamethasone, ascorbic acid, and β-glycerophosphate abbreviated as DAG (Takara Bio Inc., RRID: SCR_021372). For silencing experiments, cells were seeded into 12-well or 24-well plates with 13-mm coverslips at a density of 10,000 cells/cm^2^. At 70% confluence, we transfected with 25 nM Silencer^®^ Select siRNA (Ambion Inc., RRID: SCR_008406) using the cationic liposomes Dharma FECT siRNA Transfection Reagent III (Dharmacon, CO, United States) for 24 h. Then, the medium was changed every 2–3 days without keeping the RNA interference according to the following experimental conditions: (a) NI: L88/5 MSC in basal medium, (b) IND: L88/5 MSC in basal medium + DAG, (c) NI si *GNAS* s1/s6: L88/5 MSC in basal medium after *GNAS* exon 1 or 6 silencing, and (d) IND si *GNAS* s1/s6: L88/5 MSC in basal medium with DAG after *GNAS* exon 1 or 6 silencing. Cells were collected on days 3, 5, 7, 14, 21, and 28. Two different siRNAs were used, a custom siRNA specifically targeting Gsα exon 1 s455692 (called s1) and a predesigned siRNA s5890, targeting the common *GNAS* exon 6 (called s6), thus interfering with the transcription of all *GNAS* transcripts in addition to that of Gsα. A cross-checking to validate the first silencing with two other different custom siRNA, specifically targeting Gsα exon 1 and *GNAS* exon 6 (s455731and s4390828, respectively), was performed (**Supplementary Figure S1**). The primary sequence is available from the manufacturer (Ambion Inc.) only for the inventoried siRNA s5890, and it is freely available from PubChem (SID 160772039 - Sense siRNA Sequence: AGAUCGACGUGAUCAAGCAtt; Antisense siRNA Sequence: UGCUUGAUCACGUCGAUCUtg), at https://pubchem.ncbi.nlm.nih.gov/substance/160772039. Custom siRNAs were created by the manufacturer according to the target sequence provided by the client and were not available from the reagent manufacturer. The control performed with only the transfection reagent was validated by comparison with a scramble (sense 5′-CAAGCAACGUGAUAGAUCGtt-3′, antisense 3′-gtGUUCGUUGCACUAUCUAGC-5′) and a mismatch (sense 5′-AGACUAGCGUGUAACAGCAtt-3′, antisense 3′-gtUCUGAUCGCACAUUGUCGU-5′) (**Supplementary Figure S2**). To determine IC_50_, siRNA was transfected at concentrations from 0.01 nM to 100 nM. Then, RT-PCR assessed *GNAS* gene expression 48 h after transfection (**Supplementary Figure S3**).

### 2.2 Primary cell culture obtained from ectopic bone tissue

The ectopic ossification from the subcutaneous tissue of the PHP *GNAS*-mutated girl was collected in sterile warm PBS, and part of the tissue was embedded in the OCT and used for mineral deposit staining evaluation and immunofluorescence (IF) assays. Another part was subjected in a Petri dish for mechanical disruption in small pieces with a sterile rongeur in PBS and placed in a digesting medium composed of collagenase (2 mg/mL, Roche NimbleGen, RRID: SCR_008571), 1% trypsin, and 1% penicillin/streptomycin in Hanks’ Balanced Salt Solution (no calcium, no magnesium, and no phenol red from Thermo Fisher, RRID: SCR_008452). Then, four sequential digestion steps were performed, each 15 min long at 37°C with mild shaking, and all the pieces collected from the second to the fourth digestion were collected in the culture medium, RPMI-1640 medium containing 10% FBS (to inactivate the collagenase) and 1% penicillin/streptomycin, and then seeded in a Type I collagen-coated flask (Sigma-Aldrich, RRID: SCR_008988). After incubating at 37°C for 72 h, the supernatant containing debris and dead cells was removed, and a fresh growth culture medium without DAG was added. The RNA extraction was performed after day 7 on adherent cells following a standard protocol with TRIzol and treated with DNase I (Thermo Fisher). The gene expression was performed on the attached cells derived from the tissue without DAG to report the tissue previously characterized as faithfully as possible. The primary cells were monitored daily, evaluating at T5 and T10 the absence of *GNAS* gene expression and the degree of calcium deposition by Alizarin Red (Sigma-Aldrich, RRID: SCR_008988) staining and Alkaline Phosphatase activity (Takara Bio Inc., RRID: SCR_021372) (**Supplementary Figure S4**).

The study was performed in compliance with relevant legislation and institutional guidelines and was approved by the IRCCS Fondazione Cà Granda Ospedale Maggiore Policlinico Institutional Committee. The subjects involved in the study provided informed consent for the study.

### 2.3 *GNAS* locus imprinting stability investigation

The preservation of the normally hemimethylated status of *GNAS* differentially methylated regions (DMRs) in culture conditions and/or after the induction of OB genesis was investigated by methylation-specific multiplex ligand-dependent amplification (MS-MLPA) and the internal size standard GeneScan 500LIZ (Thermo Fisher Scientific, RRID: SCR_008452). Raw data analysis was carried out by Coffalyser v9.4 (MRC-Holland, the Netherlands).

### 2.4 cAMP assay

On days 3 and 5, intracellular basal and agonist-stimulated (10 nM PTH, Tocris Bioscience, RRID: SCR_003689) cAMP concentrations were determined with the cAMP-Glo™ Max Assay (Promega, RRID: SCR_006724) according to the manufacturer’s instructions. As a positive control, we used L88/5 cells treated with 100 μM forskolin (fsk, Sigma-Aldrich, RRID: SCR_008988). In brief, a density of 5,000/well of L88/5 cells was plated in poly-D-lysine-coated, white, clear-bottom 96-well plates (BD BioCoat, BD Biosciences, RRID: SCR_013311) with 20 mM MgCl_2_ induction buffer for 2 h. To detect luminescent signals, which were measured using a plate reader luminometer (SAFAS Flx-Xenius, Monaco), cells were incubated with cAMP Detection Solution and Kinase-Glo^®^ reagent. Data analysis to calculate the change in relative light units (ΔRLU) was performed using Microsoft Excel with linear regression. A standard curve was obtained by plotting ΔRLU values against cAMP concentrations ranging from 0 to 100 nM. The RLU values of the samples were normalized using their protein concentrations (DC Protein Assay kit, Bio-Rad Laboratories, RRID: SCR_008426).

### 2.5 Gene expression analysis

The cDNA for gene expression was obtained by the reverse transcription of 250 ng of RNA using an iScript™ cDNA Synthesis kit (Bio-Rad Laboratories, RRID: SCR_008426) according to the manufacturer’s instructions. An unbiased target-specific cDNA preamplification step was performed with SsoAdvanced PreAmp Supermix (Bio-Rad Laboratories, RRID: SCR_008426), and real-time qPCR was performed using the TaqMan™ Fast Universal PCR Master Mix and multiplexing the TaqMan^®^ RNase P assay, used as an internal reference, with one of the following TaqMan Gene expression Assays: *RUNX2* (Hs01047973_m1), Collagen IAI (*COL1A1*) (Hs00164004_m1), Alkaline Phosphatase (ALP) (Hs01029144_m1), Bone gamma Carboxyglutamate protein (*BGLAP*) (Hs01587814_g1), Integrin Binding Sialoprotein (*IBSP*) (Hs00913377_m1), and the Custom TaqMan^®^ gene expression assay designed to target Gsα exon 1 (Thermo Fisher Scientific, RRID: SCR_008452). Experiments for dentin matrix protein (*DMP1*) were carried out only on day 28 of culture using custom oligonucleotides and SsoAvdanced Universal SYBR Green Supermix (Bio-Rad Laboratories, RRID: SCR_008426) using the housekeeping gene TaqMan Gene expression Assay Hs99999905_m1 for the GAPDH gene after validation by Sanger sequencing (data not shown). Primer sequences and/or additional technical data are available upon request. Reactions were carried out in triplicate using QuantStudio3 (Thermo Fisher Scientific, RRID: SCR_008452); every time point was analyzed by the 2(−ΔC(t)) method and normalized using the NI L88/5 cells.

### 2.6 Protein quantification

Protein quantification was performed by TaqMan™ Protein Expression Assay on proximity ligation assay technology (PLA™) to detect proteins through the amplification of a surrogate DNA template. It involved three steps performed using the Protein Expression Sample Prep Kit, the TaqMan^®^ Protein Assay Open Kit, and the TaqMan™ Protein Assays Core Reagents Kit with MasterMix (Thermo Fisher Scientific, RRID: SCR_008452): (1) the binding of paired oligonucleotide-antibody probes to the target protein, (2) the ligation of oligonucleotides in proximity, and (3) the detection by qPCR with TaqMan chemistry (QuantStudio3, Thermo Fisher Scientific, RRID: SCR_008452). As a starting condition, the Gsα antibody (sc-135914, Santa Cruz Biotechnology, RRID: SCR_008987), raised against Gsα amino acid residues 11–21, after a purification step with the Melon Gel IgG Spin Purification Kit (Thermo Fisher Scientific, RRID: SCR_008452), was biotinylated with EZ-Link Sulfo-NHS-LC-Biotin (Thermo Fisher Scientific, RRID: SCR_008452). The specificity of the sc-135914 antibody was previously tested by Western blot (data not shown). HepG2 and Raji cell lysates were used as controls during the protocol setup. All reactions were carried out in triplicate, and normalized CT data using the NI L88/5 cells condition as a reference calibrator to evaluate differences in the amount of detected target protein at each time point were used.

### 2.7 Human tissue and cell staining

For calcium deposits, a surgically removed section of ectopic ossification from the subcutaneous tissue of an 18-month-old *GNAS*-mutated girl embedded in the O.C.T. compound (Sakura Finetek, CA, United States) was colored using von Kossa staining. Briefly, a 4-μm cryosection was incubated with 5% silver solution (Sigma-Aldrich, RRID: SCR_008988) and placed under ultraviolet light for 30 min. Then, the tissue was treated with 5% sodium thiosulfate (Sigma-Aldrich, RRID: SCR_008988) for 5 min and counterstained with nuclear fast red solution (Bio-Optica, Milan, Italy). Images were acquired with an Axioskop 40 microscope (Carl Zeiss Microscopy, Jena, Germany). IF studies of ectopic bone were performed using 4-μm cryosections fixed with 4% paraformaldehyde for 10 min and then permeabilized with 0.3% Triton X-100 (Sigma-Aldrich, RRID: SCR_008988) for 15 min. After blocking with 1% bovine serum albumin for 30 min at RT, tissue sections were incubated with primary antibody RUNX2 (1:50, MAB2006, R&D, RRID: SCR_013528) or Gsα (sc-135914, Santa Cruz Biotechnology, USA), washed with PBS, and incubated with secondary antibodies goat anti-rat and goat anti-mouse Alexa Fluor 488-conjugated (A-11006, A-11001, Thermo Fisher Scientific, RRID: SCR_008452) and DAPI (Sigma-Aldrich, RRID: SCR_008988) for 1 h at RT. For the analysis of L88/5 cells mineralized with ECM, Alizarin Red was used to dye calcium deposits on days 7, 14, and 21. L88/5 cells growing in 12-well plates were fixed in 70% ethanol for 10 min and incubated with 0.1% AR solution for 30 min before visualization. Images were acquired with a Zeiss Axiovert 25 inverted microscope (Carl Zeiss).

For collagen matrix, L88/5 cells growing on coverslips on days 7, 14, and 21 at each experimental condition were fixed with cold acetone for 5 min, permeabilized with 0.3% Triton X-100, and blocked with PBS containing 1% BSA at RT and then were incubated with collagen type I−FITC conjugate for 1 h (Sigma-Aldrich, RRID: SCR_008988). The IF of FGF23 (1:100, ab56326, Abcam, RRID: SCR_008987) with donkey anti-goat Alexa Fluor 546-conjugated (A-11056, Thermo Fisher Scientific, RRID: SCR_008452) and N-term Sclerostin (SOST) (1:100, # SAB1300086, Sigma-Aldrich, RRID: SCR_008988) with goat anti-rabbit Alexa Fluor 488-conjugated (#A-11008, Thermo Fisher Scientific, RRID: SCR_008452) were performed under the same conditions on day 28. The specificity of antibodies was checked by incubating with a mouse IgG1, rat IgG2b, goat IgG, or rabbit IgG Isotype Control (MA1-10405, 02-9288, 02-6202, 02-6102 Thermo Fisher Scientific, RRID: SCR_008452). All IF images were acquired with a Zeiss AxioObserver microscope equipped with a high-resolution AxioCamMRc5 digital video camera and Apotome system (Carl Zeiss Microscopy, Jena, Germany). The amount of COL1A1 and calcium deposition was quantified using the ImageJ program by drawing a region of interest (ROI).

### 2.8 Statistical analysis

Experiments were conducted on at least three replicates for each condition and time point. The comparative Ct method determined relative RNA abundance for real-time RT-PCR. Data were expressed as mean ± standard deviation (SD), and Student’s *t*-test was applied to analyze group differences. In all statistical analyses, the significance was set for *p*-values < 0.05. Analyses were performed with GraphPad Prism version 8.0.0 for Windows (GraphPad Software, San Diego, California, USA). IC_50_ (half-maximum inhibitory concentration) for *GNAS* silencing was determined using AAT Bioquest online tools (https://www.aatbio.com/tools/ic50-calculator). The number of replicates per group or condition, summary statistics, and measures of dispersion are indicated in the legend of each figure.

## 3 Results

### 3.1 Selection of crucial osteodifferentiation time points and *GNAS* haploinsufficiency induction

**Figure 1:**
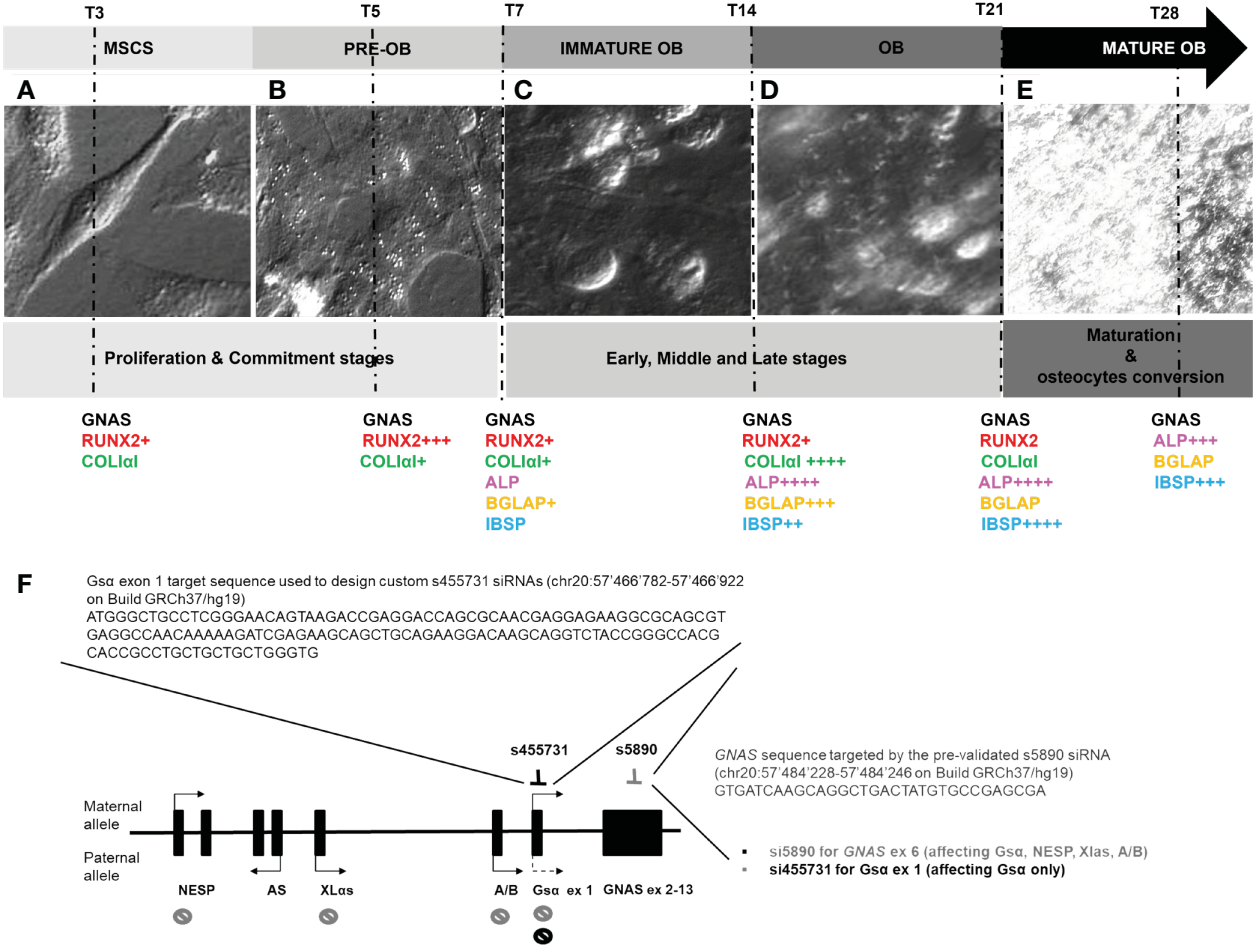
Optical images of the L88/5 MSCs main modifications from T3 to T28 during osteodifferentiation with a schematic representation of osteogenic differentiation marker expression related to the IND condition (+ = 1-fold versus NI) **(A–E)**. Representative image of the *GNAS* locus, its transcripts with relative parent-specific expression, selection of precise mRNA sequences targeting s1, which specifically silences the transcript encoding Gsα, and s6, which targets exon 6, silencing Gsα, NESP, Xlαs, and A/B transcripts **(F)**.

The daily cell morphology monitoring provided information on the crucial points to be selected for the study and confirmed by gene expression data, which were in accordance with published data. L88/5 MSC cells after DAG administration showed a fibroblast-like appearance. They actively proliferated on day 3, appearing with a flattened spheroidal form with a large cytoplasm rich in secretory vesicles, becoming preosteoblasts on day 5 (proliferation/commitment phase) (**Figures 1A, B**). On day 7, the L88/5 began to secrete ECM, becoming immature OB (early phase) (**Figure 1C**). On day 14, an intermediate step was observed, in which the L88/5 were swollen, releasing significant amounts of the matrix (middle phase) (**Figure 1C**). By day 21, the cells had a pebble-like appearance, not easily perceived due to the thick and white matrix (late stage) (**Figure 1D**). By day 28, L88/5 cells reached the complete OB maturation (maturation phase) till OS differentiation (**Figure 1E**). The time points selected were as follows: (1) day 3–5 proliferation and commitment towards the osteoblastic lineage, (2) day 7–14–21 early, middle, and late osteoblastic differentiation stage, and (3) day 28 maturation and OS conversion.

To mimic *GNAS* haploinsufficiency, we tested two different specific siRNAs, one targeting *GNAS* exon 6 (s6), affecting the expression of both Gsα and XLα transcripts, and one targeting Gsα-specific exon 1 (s1), selectively affecting the expression of the Gsα transcript (**Figure 1F**).

### 3.2 *GNAS* mRNA, Gsα expression, and cAMP activity during osteodifferentiation and after *GNAS* silencing

Before starting the analysis of osteodifferentiation markers, Gsα mRNA and protein expression and cAMP activity after the OB induction, with and without *GNAS* silencing, were first evaluated.

**Figure 2:**
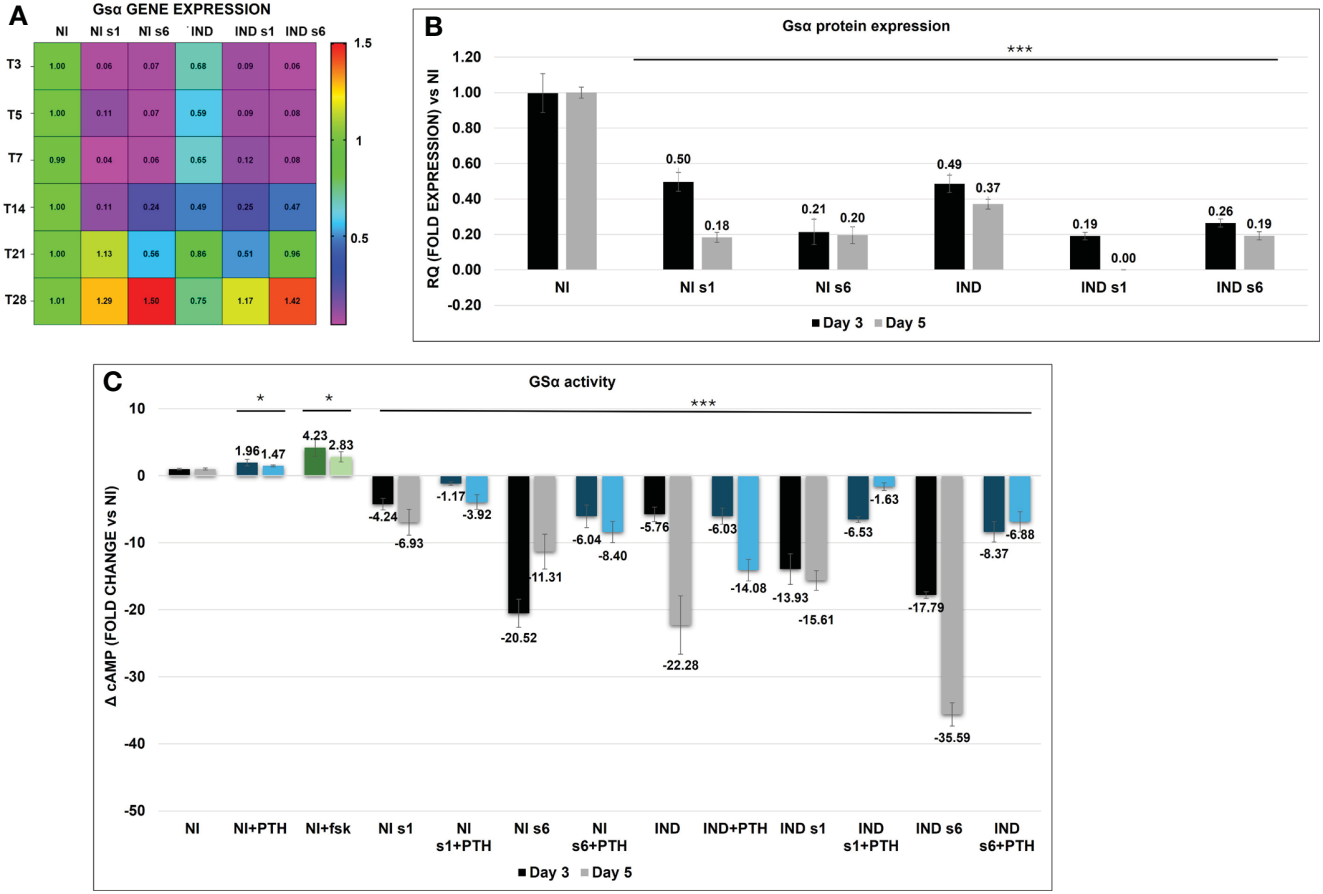
Heat map of Gsα mRNA variation in L88/5 MSCs in IND/NI with/without *GNAS* silencing condition from day 3 to 28. Expression data normalized against NI, and colored side legend for the expression **(A)**. Gsα protein expression in L88/5 MSCs in IND/NI condition with/without *GNAS* silencing from day 3 to 5 **(B)**. L88/5 MSCs intracellular cAMP levels in IND/NI with/without *GNAS* silencing condition on days 3 and 5 (black, gray). The same condition stimulates with PTH on days 3 and 5 (blue, light blue) **(C)**. fsk addition as a positive control in NI condition on days 3 and 5 (green, light green). Asterisks indicate significant differences: *p < 0.05, ***p < 0.001 by Student’s *t*-test versus related NI control. *n* = 3/group. Scale bar: 50 µm. NI, not induced L88/5 cells; NI s1 and s6, not induced L88/5 cells subject to *GNAS* siRNAs s1 and s6; IND, OB-induced L88/5 cells; IND s1 and s6, OB-induced L88/5 cells subject to *GNAS* siRNAs s1 and s6; PTH, parathyroid hormone; fsk, forskolin.

During the basic osteodifferentiation, IND cells were observed to have an initial 30% reduction in *GNAS* expression accompanied by a significant reduction in protein quantity (RQ 50% and 60% reduction on day 3 and 5, respectively) and in cAMP activity (ΔcAMP −5.75 and −22.28 on days 3 and 5, respectively) (**Figures 2A–C**). After that, *GNAS* remained downregulated, reaching the expression level of control NIs in the late differentiation phase (days 21–28). The analysis of both s1 and s6 silenced cells, both INDs and NIs, revealed a significant Gsα downregulation till day 14 after DAG administration, with an efficiency of more than 90% up to day 7. These last data were also validated by the second pair of on-target oligonucleotides against vs Gsα ex 1 and *GNAS* ex 6 (**Figure 2A**; **Supplementary Figure S1**). The cAMP remained downregulated, and the activity was activated only in NI by the addition of fsk and/or PTH (**Figure 2C**). Though we performed all experiments using both siRNAs, considering that we did not observe any significant difference in the expression of Gsα between S1 and S6, for simplicity and brevity purposes, we chose to report the mean of the results obtained. However, figures report raw numerical data. MS-MLPA analysis of the differential methylation at *GNAS* DMRs at different time points confirmed that *GNAS* imprinting was stable and that *GNAS* mRNA expression was unaffected by imprinting alterations (**Supplementary Table S1**).

### 3.3 Osteodifferentiation markers trend: *RUNX2, COL1A1, ALPL, BGLAP*, and *IBSP*

**Figure 3:**
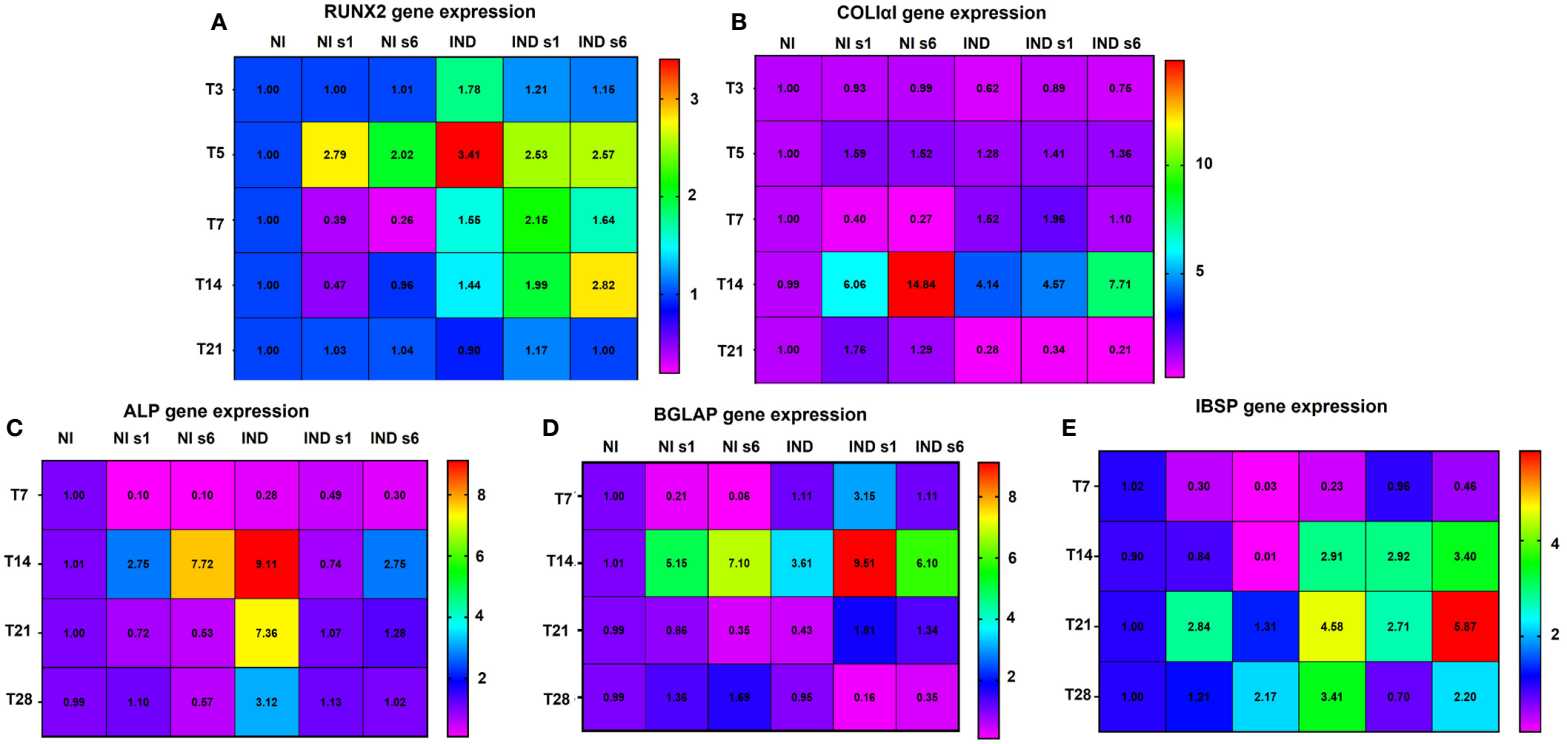
Multipanel heat map summarizing the gene expression variation of *RUNX2* **(A)**, *COLIαI* **(B)**, *ALP* **(C)**, *BGLAP* **(D)**, and *IBSP* **(E)** in L88/5 MSCs in IND/NI condition with/without *GNAS* silencing from day 3 to 28. Expression data are normalized against NI for each time point. NI, not induced L88/5 cells; NI s1 and s6, not induced L88/5 cells subject to *GNAS* siRNAs s1 and s6; IND, OB-induced L88/5 cells; IND s1 and s6, OB-induced L88/5 cells subject to *GNAS* siRNAs s1 and s6.

The expression of *RUNX2*, the principal gene responsible for the commitment of MSCs to OB lineage, was first analyzed (**Figure 3A**) (5). While *RUNX2* expression increased immediately after stimulation, reaching its maximum on day 5 in IND cells (IND RQ ~ 1.78 and 3.4, respectively), in the silenced condition, its expression increased only on day 5 (si*GNAS* IND RQ ~2.55, si*GNAS* NI RQ ~ 2.40). During the early and middle stages, from day 7 to 14, *RUNX2* remained upregulated in both IND conditions (IND RQ ~ 1.55/1.44 and si*GNAS* IND RQ ~ 1.89/2.40, respectively), returning to basal levels during late and maturation phases (**Figure 3A**).

The *COL1A1*, the main organic component of the bone matrix synthesized by OB before mineralization and strongly promoting proliferation, survival, adhesion, and osteodifferentiation, was then analyzed (34). *COL1A1* expression increased on day 7 only in inducted and silenced cells (IND RQ ~ 1.52, si*GNAS* IND RQ ~ 1.53), reaching a maximum peak at T14 (IND RQ ~ 4.14, si*GNAS* IND RQ ~ 6.14). A remarkable increase of non-inducted silenced cells occurred on day 14 (si*GNAS* NI RQ ~10.45). A sharp drop happened in all conditions in the late and mature phases (**Figure 3B**).

We then proceeded to analyze *ALPL*, which hydrolyzes pyrophosphate, producing inorganic phosphate useful to promote mineralization (35). *ALPL* suddenly increased in the middle stage on day 14 in all conditions (IND RQ ~ 9.11, si*GNAS* IND RQ ~ 1.75, si*GNAS* NI RQ ~5.23), followed by a sharp reduction during late and mature stages, except for INDs (IND RQ ~ 7.36/3.12 respectively) (**Figure 3C**).

Therefore, we moved to analyze *BGLAP*, secreted solely by OBs, representing the most abundant non-collagenous protein of the matrix, with a high affinity for calcium ions and apatite crystal hydroxyapatite, and considered as a middle/late marker of bone formation (36). Accordingly, *BGLAP* was significantly increased only in the middle stage on day 14 in all conditions, and mostly in silenced cells (RQ IND ~ 3.61, si*GNAS* IND ~ 7.80, si*GNAS* NI ~ 6.12), followed by a sharp fall to basal conditions (**Figure 3D**).

Lastly, the expression of *IBSP*, a member of the SIBLING family of proteins, which are markers of mineralization onset of osteodifferentiation functionality, was examined (37). In all conditions of IND cells, the *IBSP* expression increased during the middle and late stages, reaching its apex on day 21 and remaining upregulated during the maturation phases (RQ IND 2.91/4.58/3.41 and RQ IND si*GNAS* 3.16/4.29/1.45, respectively). A slight activation occurred in the NI condition in the later phase, on days 21–28 (RQ si*GNAS* 2.07/1.69, respectively) (**Figure 3E**).

### 3.4 Matrix formation: COLIαI and calcium deposition

After the commitment phase, cells deposited mineralized ECM, which was composed of an organic fraction, mainly represented by *COL1A1*, and a crystalline fraction of inorganic hydroxyapatite and accessory structural proteins (38).

**Figure 4:**
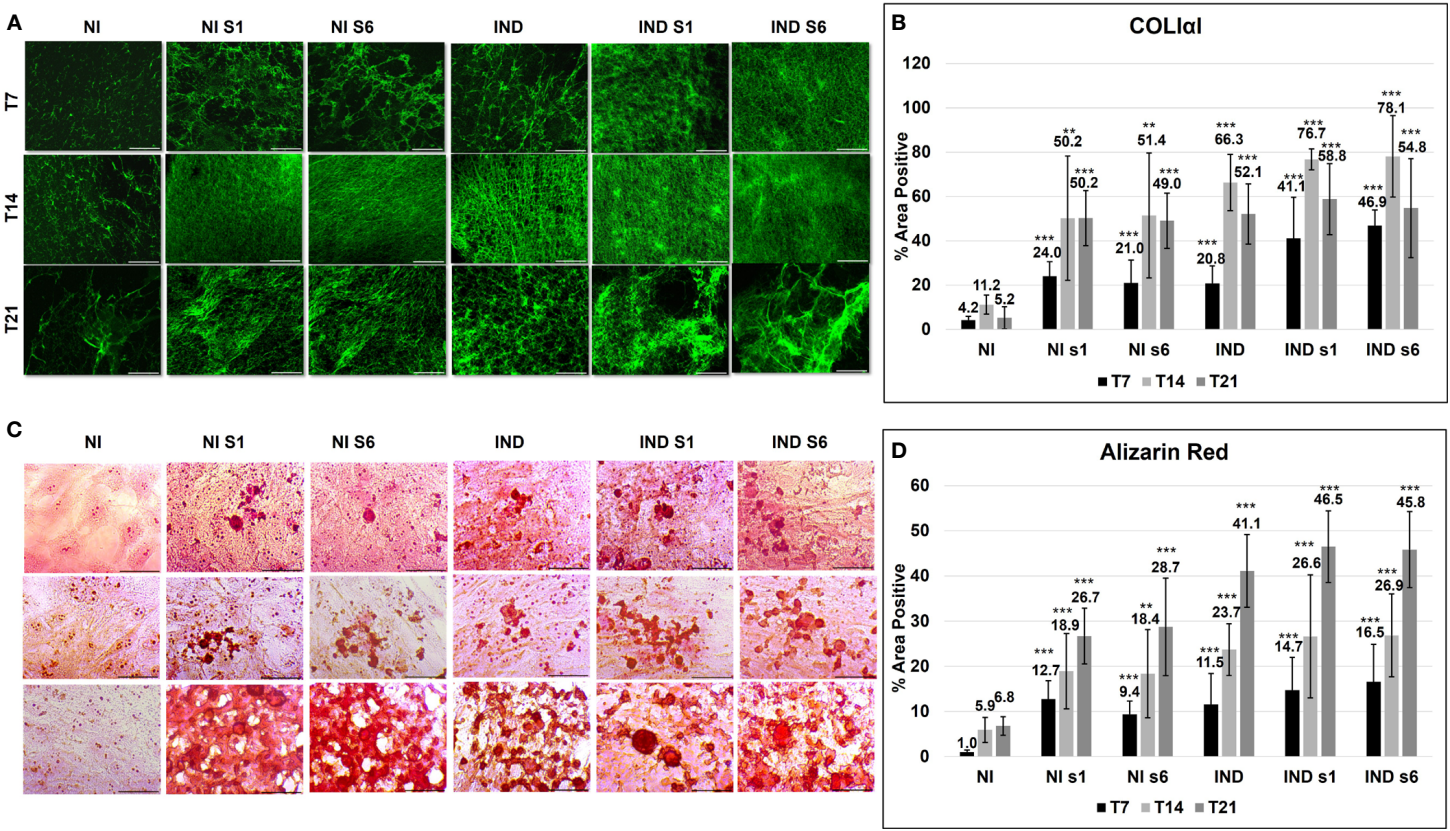
ECM deposition evaluated by COLIαI IF (green) **(A, B)** and Alizarin red **(C, D)** in L88/5 MSCs in IND/NI with/without *GNAS* silencing condition from day 7 to 21. Asterisks indicate significant differences: **p < 0.01, ***p < 0.001 by Student’s *t*-test versus related control NI. *n* = 9. Scale bar: 50 µm. NI, not induced L88/5 cells; NI s1 and s6, not induced L88/5 cells subject to *GNAS* siRNAs s1 and s6; IND, OB-induced L88/5 cells; IND s1 and s6, OB-induced L88/5 cells subject to *GNAS* siRNAs s1 and s6.

The initial formation of the *COL1A1* network was visible on day 7 in all conditions compared to NI (si*GNAS* NI ~ 22.5, IND 20.8, si*GNAS* IND ~ 44). Further collagen deposition resulted in a strong and spatially well-regulated network on day 14 (si*GNAS* NI ~ 50.8, IND 66.3, and si*GNAS* IND ~ 77.4) (**Figures 4A, B**). Then, on day 21, the collagen matrix changed its appearance and acquired a mature hollow organized area in the IND condition only, whereas a messy mass was observed in silenced conditions The quantity remained almost stable with a non-significant decreasing trend on day 21 in all conditions (si*GNAS* NI ~ 49.6, IND 52.1, and si*GNAS* IND ~ 56.8) (**Figures 4A, B**). In all silenced conditions, a more consistent deposition was observed, with a denser mesh due to the overlapping of different fibrils layers but characterized by a disordered grid. No quantitative COL1A1 was detected, but only a qualitative difference in mesh deposition was detected comparing the same time point from S1 and S6 (**Figures 4A, B**).

The hydroxyapatite crystals were deposited within the ECM matrix, expanding thanks to an adequate supply of extracellular calcium (38) (**Figure 4C**). On day 7, all conditions exhibited small intracellular mineralization foci. Such foci expanded on days 14 and 21. In particular, on day 21, a visible morphological difference between IND and NI/IND si*GNAS* appeared: a well-defined and organized mineralized ECM in the first against a widespread, disordered, and overloaded one in the second (**Figure 4C**). The calcium deposition amount increased significantly since day 7 in all conditions compared to NI (si*GNAS* NI ~ 11, IND 11.5, si*GNAS* IND ~ 15.6) and day 14 (si*GNAS* NI ~ 18.6, IND 23.7 and si*GNAS* IND ~ 26.7) with a maximum peak on day 21 in all conditions (si*GNAS* NI ~ 27.7, IND 23.7, si*GNAS* IND ~ 46). Also in this case, no quantitative difference in calcium deposition was detected between S1 and S6 at the same time point (**Figure 4D**).

### 3.5 Osteocyte markers: *DMP1, FGF23*, and *SOST*

**Figure 5:**
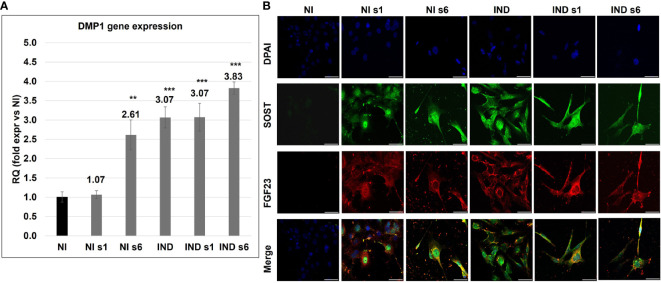
Gene expression variation of *DMP1* **(A)** and double IFI of FGF23 (red) and SOST (Green) in L88/5 MSCs **(B)** in IND/NI condition with/without *GNAS* silencing on day 28. DAPI in blue. Asterisks indicate significant differences: **p < 0.01, ***p < 0.001 by Student’s *t*-test versus related NI control. *n* = 3/group. Scale bar: 50 µm. NI, not induced L88/5 cells; NI s1 and s6, not induced L88/5 cells subject to *GNAS* siRNAs s1 and s6; IND: OB-induced L88/5 cells; IND s1 and s6, OB-induced L88/5 cells subject to *GNAS* siRNAs s1 and s6.

After acquiring a mature OB phenotype and creating the mineralized ECM, cells are involved in the regulation, remodeling, and maintenance of bone, and a proportion of them differentiate into OS. The progression to OS can be tracked morphologically and through the temporally selective expression of several endogenous genes, *DMP1*, *FGF23*, and *SOST*. Analyzing *DMP1*, mainly produced by mature OS and playing a key role in maintaining bone mineralization and controlling phosphate homeostasis, we observed an upregulation in all conditions on day 28 (IND RQ ~ 3.07, si*GNAS* IND 3.45, si*GNAS* NI 1.84) (**Figure 5A**) (39). Then, during the analysis of the OS markers FGF23 and SOST, a positive IF signal was spread in all examined conditions on day 28, with a more marked signal in si*GNAS* IND, where a stellate OS-like shape confirmed the OS transition (**Figure 5B**) (40, 41).

### 3.6 Analysis of a surgically removed sample of ectopic ossification from a PHP patient

**Figure 6:**
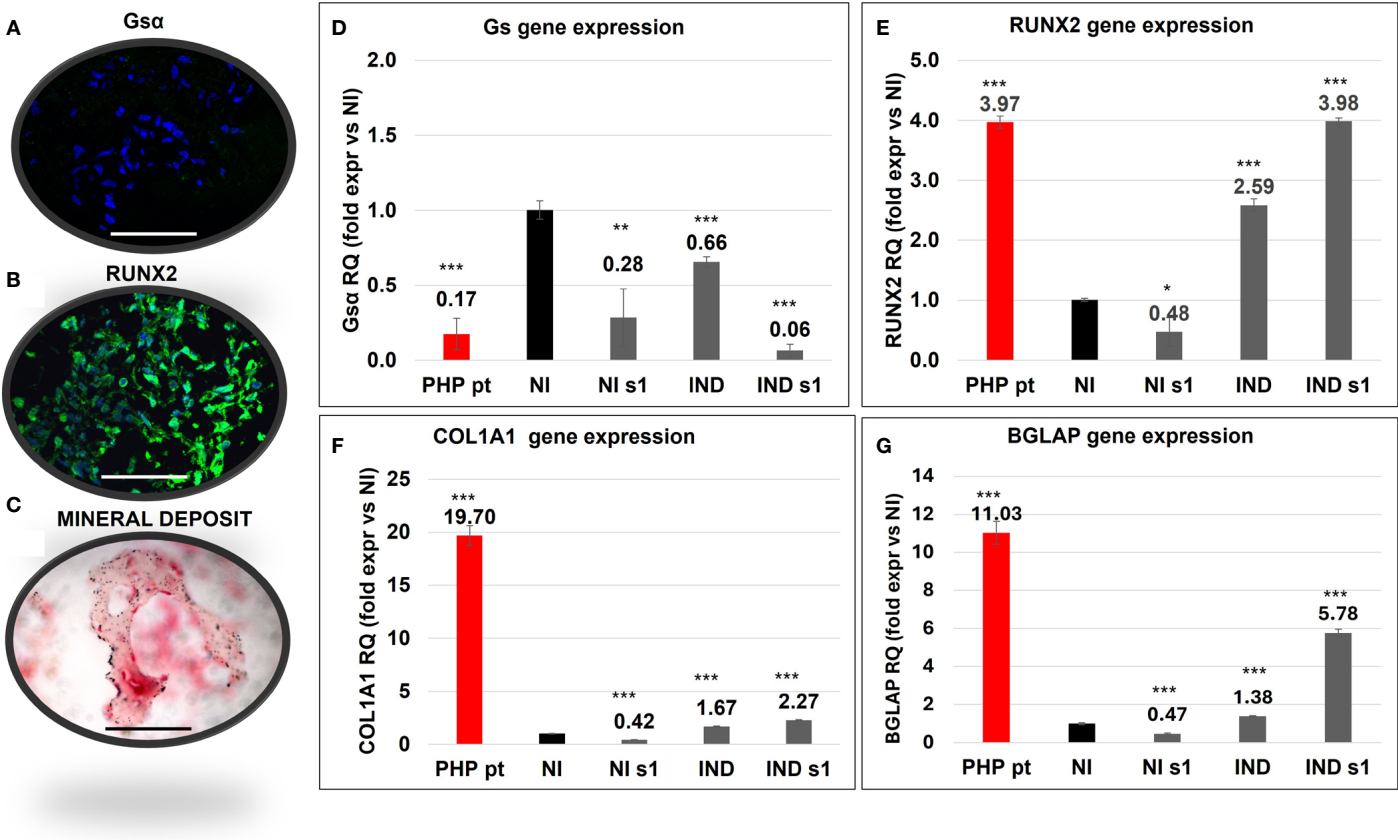
Characterization of a section of ectopic bone formation surgically removed from a *GNAS*-mutated patient (PHP pt) evaluated by Gsα **(A)** and RUNX2 **(B)** IF, and Von Kossa staining **(C)**. Comparison of the gene expression of Gsα **(D)**, *RUNX2* **(E)**, *COL1A1* **(F)**, and *BGLAP* **(G)** between PHP pt-derived cells and in L88/5 MSCs in IND/NI with/without *GNAS* silencing conditions on day 10. Asterisks indicate significant differences: *p < 0.05, **p < 0.01, ***p < 0.001 by Student’s *t*-test versus related NI control. *n* = 3/group. Scale bar: 50 µm. NI, not induced L88/5 cells; NI s1, not induced L88/5 cells subject to *GNAS* siRNAs s1; IND, OB-induced L88/5 cells; IND s1, OB-induced L88/5 cells subject to *GNAS* siRNAs s1.

A surgically removed ectopic ossification from the subcutaneous tissue of an 18-month-old *GNAS*-mutated girl was characterized. IF demonstrated the reduction of Gsα, as expected, being the girl affected by a *GNAS* mutation and the presence of *RUNX2* expression (**Figures 6A, B**). The Von Kossa staining highlighted mineral deposits, and the counterstaining with nuclear fast red solution allowed the identification of both OBs and OSs (**Figure 6C**).

We compared the behavior of our *in vitro* model against the one seen in ectopic bone cells. In particular, *GNAS*, *RUNX2*, *COL1A1*, and *BGLAP* gene expression in the PHP sample was compared with that of L88/5 MSCs, subject to different experimental conditions, on day 10. In accordance with the presence of a deleterious *GNAS* variant and subsequent haploinsufficiency, Gsα mRNA was reduced (RQ PHP pt ~ 0.17) in the PHP case as in our silenced cell model (RQ si*GNAS* NI 0.28 and si*GNAS* IND ~ 0.06) (**Figure 6D**). *RUNX2* expression, upregulated in the PHP case (RQ PHP pt ~ 3.97), was upregulated in IND and, mainly, in si*GNAS* IND (RQ IND ~2.59/~3.98, respectively) (**Figure 6E**). *COL1A1* followed the same trend in both IND and si*GNAS* IND, even though it was higher in the patient (RQ PHP pt ~ 19.70 vs. RQ IND 1.67 and si*GNAS* IND ~ 2.27). *BGLAP* was overexpressed in the PHP patient (RQ PHP pt ~ 11.03) and upregulated in IND and si*GNAS* IND (RQ IND ~1.38 and si*GNAS* IND ~ 5.78), while no expression was found in any NI condition (**Figures 6F, G**).

## 4 Discussion and conclusion

Most *GNAS*-based disorders share the common feature of *de novo* formation of islands of ectopic bone. Data from studies in transgenic mice support the concept that *GNAS* is a crucial regulator of the lineage switching between OB and adipocyte fates in precursor cells and that its role may be to prevent aberrant osteoinduction (25–28). However, the pathophysiological mechanisms by which *GNAS* alterations lead to HO in humans are not fully understood.

Firstly, considering that the simple MSC osteodifferentiation is a minefield, for good standardization, we decided to apply the widely used DAG protocol, in which each component contributes to creating an intricate machine interconnected and cooperating towards osteoblastic differentiation. The careful daily cell monitoring, and the osteoblastic markers analysis provided precious information about the potential of L88/5 MSC to differentiate into OB, confirming that the process takes approximately 28 days and is characterized by a specific phase sequence, which allowed us to set up time points for subsequent analysis (42).

Secondly, we proved the efficiency of our PHP *in vitro* model represented by the *GNAS* silencing on exon 1 (affecting selectively the Gsα transcript) or exon 6 (affecting both Gsα and XLα transcripts). In both cases, we obtained more than 90% efficiency up to day 7, supported by the reduction of Gsα protein expression and of cAMP production by the adenylyl cyclase, even under PTH agonist stimulation. The goodness of experiments was also validated by the increased production of cAMP in basal cells treated with PTH or fsk (28). Moreover, as previously described, the absence of Gsα expression was detected during osteodifferentiation in our silenced cells (22).

Considering that the bone is formed by the activation of a highly hierarchical gene pathway, we started our analysis from *RUNX2*, the master conductor of osteodifferentiation. It is crucial for the commitment phase of MSCs and orchestrates the transcription of proteins involved in the development and maintenance of bone (43). In accordance with Lietman and colleagues, our data showed that reducing the Gsα expression increased the expression of *RUNX2* in L88/5 MSCs, in both IND and NI conditions (25). Therefore, subsequent cAMP activity reduction could lead to abnormal osteodifferentiation in patients affected by PHP, AHO, POH, and osteoma cutis (30).

According to *RUNX2*’s role as a positive regulator of osteogenesis, which activates the tightly regulated expression of OB-specific genes, in our *GNAS*-silenced NI and IND cells, OB markers (*COL1A1*, *BGLAP*, *ALPL*, and *IBSP*) were generally upregulated during the middle and late stages, with different time point trends.

To further understand the influence of *GNAS* silencing on osteodifferentiation, we examined the matrix formation and mineralization, that is, the environment responsible for cell adhesion, proliferation, and response to growth factors, and the basis of physiological bone turnover (44). Despite many minor collagen proteins, *COL1A1* is the main constituent of ECM. Its relevance was demonstrated by the absence of osteodifferentiation when cells were cultured with collagenase and by the presence when they were cultivated with ECM without DAG (45). Collagen is a triple-strand associated with fibrils through intra/intermolecular bonds of hydroxyproline and lysine with a regular arrangement, which, once impaired, can lead to bone malformations (46). Many studies report that a post-translational modification of *COL1A1* seriously compromises the properties of the fibrillar helix and is the basis of several skeletal pathologies (imperfect osteogenesis and osteoporosis) (47, 48). In accordance with Brewer’s studies on *GNAS* null mice, we observed significant *COL1A1* deposition in both *GNAS*-silenced cell conditions. This increased deposition should not be underestimated, considering that an increased ECM release could trigger the MAPK signaling pathway, leading to *RUNX2* transcription through the integrin α2β1 (32, 49). In addition, an abnormal conformation of the deposited fibril was observed. In fact, while IND cells exhibited an orderly mesh with well-defined and distinct thick fibers, in contrast, all silenced conditions showed a finer overlapping mesh, messy and narrow. Considering the relationship between the collagen fibril diameter and the Ca/P ratio, we were not surprised by the different deposition of calcium observed between the conditions analyzed (46).

Recent reports suggested an additional role for *GNAS* signaling: a regulator of OS function (39). Mice with conditional ablation of Gsα had an osteopenic phenotype, and an increased number of OS characterized their cortical bone with disorganized lacunar networks and high *SOST* expression (50). Moreover, previous studies reported that pre-OB embedded in a highly condensed structure is more efficient in triggering the evolution into OS-like cells, demonstrating that the transition of immature OB to OS may occur at an early stage of bone development. In our *GNAS*-silenced cells, the significant downregulation of *RUNX2*, after the acquisition of a mature phenotype, might collaborate with the highly embedding mineralized ECM, in triggering a more premature “aging” towards OS. We hypothesized that the increased matrix deposition in silenced cells favored, since the early immature phase, the acquisition of an OS-like phenotype. The takeover towards OSs was further supported by the increase in *DMP1* mRNA, an early marker for young OSs, crucial for the initial development of dendrites. Afterwards, a strong expression of *SOST*, which is a negative regulator of bone formation negatively regulated by the Gαs-PKA-mediated signaling, was visible. This result was also corroborated by the upregulation of the OS marker *FGF23* (41, 51).

Then, our study shows that (1) *GNAS* haploinsufficiency alone induces osteodifferentiation of NI cells, and (2) the simultaneous presence of induction accelerates the process towards an OS-like phenotype. Our data confirm the already-known role of Gsα in preventing ectopic osteoblastogenesis due to its crucial role in orchestrating the Wnt and Hh signaling pathways (52). Gsα enhances the canonical Wnt pathway, promoting the MSC progenitor to enter the OB lineage and inhibiting its complete differentiation and all OBs already channeled into the osteoblastic lineage. Furthermore, Gsα inhibits the Hh pathway, which usually promotes the entry of the MSC progenitor into the OB lineage and its complete differentiation (by producing cAMP and activating PKA) (53). In our *GNAS-*silenced cells, the regulation of the Wnt and Hh pathways is reversed, providing a sensitized background for accelerated differentiation of OBs into OSs.

We also observed that the simultaneous presence of DAG accelerates the OB’s differentiation into OSs containing components aimed at good regulation of osteodifferentiation. In short, dexamethasone activates *RUNX2* to recruit MSCs into the OB lineage via its co-activators (FHL2/LEF1-TCF/TAZ). This mechanism is aided by a BMP2 signal upstream activated by the WNT pathway and released from both human stromal bone marrow and pre-OB activating the Smad and Ras/MAPK/AP-1 pathway regulating the *RUNX2* transcription and its co-activators (p300, CBP) (54–57). Ascorbic acid is an additional player stimulating the production of the ECM molecules, which bound to pre/OB via α2β1 integrins and activates the MAPK signaling pathway and thus the transcription of *RUNX2* (49). Finally, beta-glycerol phosphate, whose hydrolysis by ALP leads to the release of Pi by reacting with calcium ions, is involved in the final process of matrix mineralization (58). Then, it is unsurprising that the inactivation of *GNAS* combined with DAG accelerates the process toward an OS-like phenotype.

Another important concept is that although no difference was observed in the expression of Gsα between S1 and S6, some differences between osteodifferentiation markers in S1 and S6 were observed in both NI and IND, with a different timing depending on the role of the marker during osteogenesis. In some cases, a prominent effect seems to be evident in siGNAS S6, and we can only surmise that the significant impact of S6 can be attributed to the silencing of Gsα, NESP, and XLα compared to Gsα alone in the case of S1. However, looking more closely at each marker, this was true for RUNX2 in IND S6, for COLIαI and ALP in both NI and IND S6, for BGLAP only in NI S6 (in S1 IND is the opposite), and finally for IBSP in IND S6. Although we do not have an explanation for these subtle specific differences, we believe that the main result is that GNAS haploinsufficiency alone induces osteodifferentiation and is accelerated by DAG toward the OS, and this is true under all silencing conditions but with a different time scale depending on the conditions. Nevertheless, supported by the absence of quantitative and qualitative differences in matrix formation and calcium deposition between S1 and S6 underlying the pathology, we decided to maintain the average of the markers for ease of reading.

Finally, we analyzed the RNA extracted from a surgical sample of heterotopic bone derived from an 18-month-old child who inherited a *GNAS-*inactivating variant from the affected mother. As expected, the Gsα transcript was downregulated, *RUNX2*, *COL1A1*, and *BGLAP* were upregulated, and an abnormal mineral deposition was present. The gene expression pattern of mutated cells was comparable to our si*GNAS* IND cells, with bone markers substantially upregulated on day 10. This result further supports that our *GNAS*-silenced cells could be considered a good *in vitro* model to investigate the effects on osteogenesis of *GNAS* haploinsufficiency.

To conclude, our data represent the initial part of our research aimed at demonstrating that our inducted *GNAS*-silenced cells represent a reliable human *in vitro* model to investigate the pathophysiology of *GNAS*-related ectopic ossifications in PHP, AHO, and POH. The topic of the present study is based on a challenging, rare disease whose investigation lacks an established cell model. Despite the limitations of having data from primary cells and tissue from only a patient, our data support the potential of *GNAS*-silenced L88/5 MSCs as an *in vitro* model for elucidating the molecular mechanisms involved in ectopic bone formation in PHP disease. A deeper analysis of each osteodifferentiation marker supported the hypothesis that ectopic bone formation is not exclusively a cell-mediated process and requires a combination of *GNAS* inactivation and changes in the extracellular matrix environment. Finally, our result showed that the abnormal matrix deposition of *GNAS*-silenced cells could be involved in the stimulation of OBs toward premature “aging” toward OSs.

## Data availability statement

The original contributions presented in the study are publicly available. This data can be found here: https://figshare.com/; 10.6084/m9.figshare.24072435.

## Ethics statement

The studies involving humans were approved by IRCCS Fondazione Cà Granda Ospedale Maggiore Policlinico Institutional Committee. The studies were conducted in accordance with the local legislation and institutional requirements. Written informed consent for participation in this study was provided by the participants’ legal guardians/next of kin.

## Author contributions

FE: Conceptualization, Data curation, Project administration, Supervision, Writing – original draft, Formal analysis, Funding acquisition, Investigation, Validation, Visualization, Writing – review & editing. DM: Conceptualization, Data curation, Supervision, Writing – original draft, Formal analysis, Investigation, Validation, Visualization, Writing – review & editing. MI: Investigation, Validation, Writing – review & editing. FB: Investigation, Validation, Writing – review & editing. MM: Investigation, Validation, Writing – review & editing. GD: Formal analysis, Visualization, Writing – review & editing. AP: Formal analysis, Visualization, Writing – review & editing. CL: Investigation, Validation, Writing – review & editing. PM: Funding acquisition, Resources, Writing – review & editing. MA: Resources, Writing – review & editing, Funding acquisition. GC: Funding acquisition, Writing – review & editing, Resources. CA: Resources, Writing – review & editing, Visualization. GM: Funding acquisition, Resources, Writing – review & editing, Visualization.
