# Supplementary material for: Targeted silencing of GNAS in a human model of osteoprogenitor cells results in the deregulation of the osteogenic differentiation program
Source: Front Endocrinol (Lausanne). 2024 May 17;15:1296886. doi: 10.3389/fendo.2024.1296886 (PMC11140044; doi:10.3389/fendo.2024.1296886)
Supplement: Supplementary file 1 [file DataSheet_1.pdf]

Supplementary Material

Figure S1

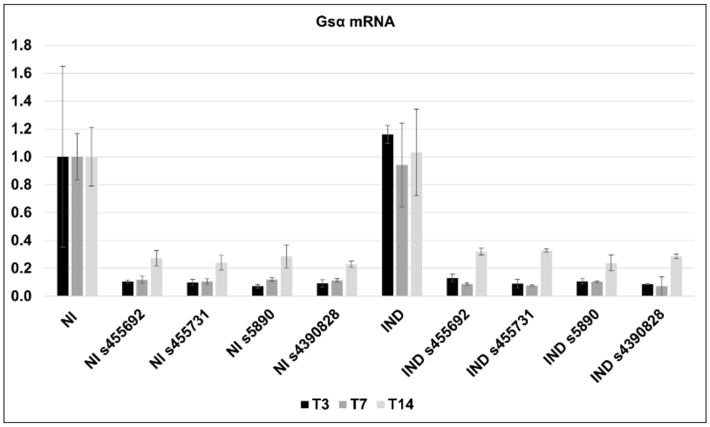

Fig S1: Gsα mRNA expression in L88/5 cells NI, IND conditions at T3, 7, 14 Day subjected to siRNA (s455731 and s455692) vs Gsα ex 1 affecting Gsα only and siRNA (s5890 and s4390828) vs *GNAS* ex 6 affecting Gsα, NESP, Xlas, A/B. n=3/group

Figure S2

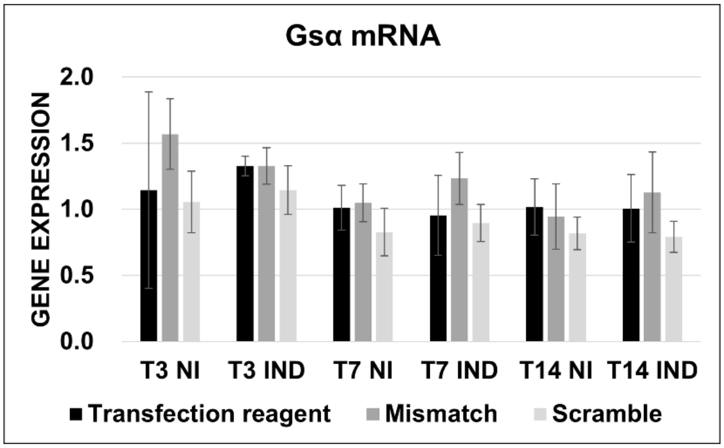

Fig S2: Gsα mRNA expression in L88/5 cells NI, IND conditions at T3, 7, 14 Day subjected to the only transfection reagent, mismatch, and scramble of siRNA (s5890) vs *GNAS* ex 6 affecting Gsα, NESP, Xlas, A/B. n=3/group

Figure S3

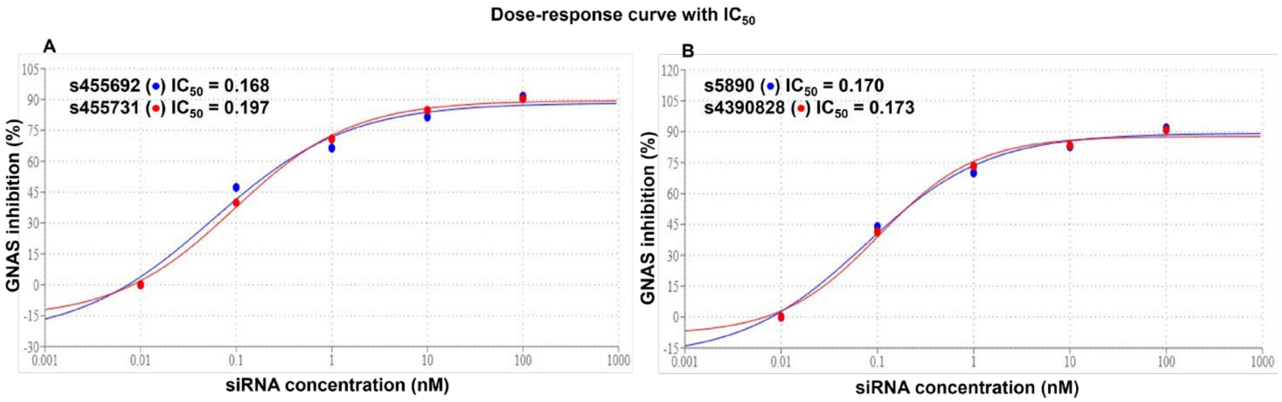

Fig S3: IC50 (half maximum inhibitory concentration) for *GNAS* silencing using L88/5 cells transfected with siRNA (s455731 and s455692) vs Gsα ex 1 affecting Gsα only **(A)** and siRNA (s5890 and s4390828) vs *GNAS* ex 6 affecting Gsα, NESP, Xlas, A/B **(B)** at the concentrations indicated at 48 h. The results were given as the mean of triplicate experiments. *GNAS* gene expression was determined using AAT Bioquest online tools (https://www.aatbio.com/tools/ic50-calculator).

Figure S4

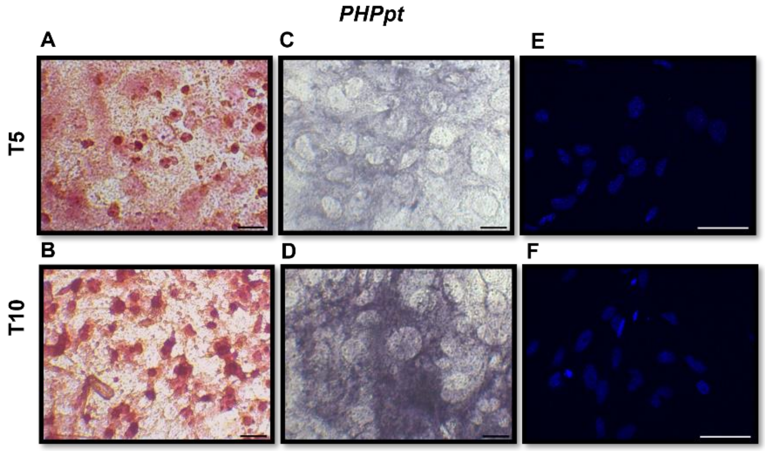

Fig S4: Alizarin red (A, B), ALP (C, D), and Gsα with DAPI in blue (E, F) staining on cells extracted from ectopic bone formation surgically removed from a GNAS-mutated patient (PHP pt) on Days 5 and 10. Scale bar: 50 µm.

Table S1: Differential methylation stability at GNAS DMRs and Gsα biallelic expression

Sample name	T0	T21 NI	T21 IND
**Gene**	**Ratio**	**Ratio**	**Ratio**
**STX16 Ex01 Digestion ctrl**	0.1	0.008	0.06
**STX16 Exon 03**	1.01	0.91	1.01
**STX16 Exon 05**	1.04	1.07	0.94
**STX16 Exon 06**	0.93	1.11	1.06
**STX16 Exon 08**	1	1	0.94
**NESP Exon 01A**	**0.58**	**0.61**	**0.55**
**NESP Exon 01B**	**0.44**	**0.48**	**0.54**
**NESP Exon 01C**	**0.62**	**0.6**	**0.58**
**NESPAS Exon 01A**	**0.48**	**0.58**	**0.47**
**NESPAS Exon 01B**	**0.41**	**0.63**	**0.51**
**NESPAS intron 01**	**0.6**	**0.68**	**0.52**
**GNAS XLExon 01A**	**0.6**	**0.38**	**0.5**
**GNAS XLExon 01B**	**0.63**	**0.51**	**0.61**
**GNAS XLExon 01C**	**0.49**	**0.5**	**0.51**
**GNAS XLExon 01D**	**0.45**	**0.44**	**0.43**

**GNAS A/B Exon 01A**	**0.53**	**0.49**	**0.5**
**GNAS A/B Exon 01A**	**0.63**	**0.52**	**0.68**
**GNAS Exon 01A Digestion ctrl**	0.1	0.11	0.09
**GNAS Exon 01B Digestion ctrl**	0.11	0.12	0.14
**GNAS Exon 02**	1.02	1.15	1.07
**GNAS Exon 03**	1.1	0.96	1.06
**GNAS Exon 04**	0.95	1.06	1.02
**GNAS Exon 06**	0.91	1.01	0.96
**GNAS Exon 07**	0.97	0.8	0.89
**GNAS Exon 09**	1.08	1.04	0.98
**GNAS Exon 11**	0.84	0.8	0.88
**GNAS Exon 13**	1.17	1.21	0.8
**Digestion ctrl**	0	0	0
**Digestion ctrl**	0	0	0

Tab S1: Representative table shows GNAS DMRs methylation ratios by the MS-MLPA analysis. The first column shows the probes' genomic location, according to their physiological order on chr20. The methylation ratio of ADMSCs at day 0 is reported in the second column. The last columns show methylation ratios determined in ADMSCs after 21 days of culture with or without the osteogenic differentiation medium. To interpret MS-MLPA data, we underline that only a subset of probes contains the restriction site and are used to calculate methylation ratios. This translates into ratios

around 1 for probes without the restriction site or methylation probes in completely methylated areas, while values about 0.5 or 0 for hemimethylated or unmethylated sites, respectively. Digestion ctrl = probes targeting sequences not subject to DNA methylation used to confirm the successful enzymatic digestion by the methylation-sensitive Hha I. The light grey color identifies methylation-sensitive probes. Bold values highlight methylation ratios for GNAS DMRs.
